# 

**Published:** 1849-03

**Authors:** 


Dr. Morton's new and splendid work on Anatomy, will be noticed in our next No.; also Graves' Clinical Medicine, Gooch on females (which have just been received), and other new publications.

